# Overall Survival and Complication Rates in the Treatment of Liver Carcinoma: A Comparative Study of Ultrasound, Computed Tomography, and Combined Ultrasound and Computed Tomography Guidance for Radiofrequency Ablation

**DOI:** 10.3390/diagnostics15141754

**Published:** 2025-07-11

**Authors:** Chia-Hsien Chien, Chia-Ling Chiang, Huei-Lung Liang, Jer-Shyung Huang, Chia-Jung Tsai

**Affiliations:** 1 Department of Radiology, Kaohsiung Veterans General Hospital, Kaohsiung City 813, Taiwan; gueensy@gmail.com; 2 Division of Interventional Radiology, Department of Radiology, Kaohsiung Veterans General Hospital, Kaohsiung City 813, Taiwan; clchiang1104@vghks.gov.tw (C.-L.C.); jshaung@vghks.gov.tw (J.-S.H.); 3 Division of Abdominal Radiology, Department of Radiology, Kaohsiung Veterans General Hospital, Kaohsiung City 813, Taiwan; hlliang@vghks.gov.tw; 4 Department of Medical Imaging and Radiological Sciences, I-Shou University, Kaohsiung City 824, Taiwan

**Keywords:** radiofrequency ablation, US-guided, CT-guided, US/CT-guided, HCC

## Abstract

**Background:** Liver cancer is a major health concern worldwide. Radiofrequency ablation is a safe treatment option that can be guided by either ultrasound, computer tomography (CT), or fluoroscopy. Although ultrasound-guided radiofrequency ablation is commonly used in clinical practice, radiofrequency ablation guided by CT is more precise but requires more time and does not offer real-time monitoring, which may result in complications such as pneumothorax or organ damage. **Objectives:** In this study, we investigated the effect of ultrasound, CT, and combined ultrasound/CT guidance on patient survival and complication development. **Methods:** A total of 982 radiofrequency ablation sessions conducted on 553 patients were analyzed. Clinical outcomes were assessed during follow-up to determine the survival and recurrence rates of malignant tumors. **Results:** Overall, the three guidance approaches exhibited significant differences in terms of tumor size, number, complication development, and treatment duration. However, no significant differences were observed in survival rate. A comparison of the effect of CT guidance and ultrasound guidance on complication development revealed a higher odds ratio for CT guidance in some cases. A comparison of combined ultrasound/CT guidance and ultrasound guidance revealed nonsignificant differences in complication development. A comparison of CT guidance and combined ultrasound/CT guidance revealed a higher odds ratio for CT guidance in some cases. Radiofrequency ablation is a safe and effective treatment for liver tumors. However, CT has an increased incidence of complications. **Conclusions:** Combined ultrasound/computer tomography guidance is recommended for patients with multiple or large tumors or tumors near the hepatic dome or diaphragm.

## 1. Introduction

According to the 2020 World Cancer Report by the World Health Organization, liver cancer is the sixth most prevalent cancer in the world, with the third-highest mortality rate following lung and colorectal cancers. Liver tumors are treated using either curative or palliative methods. These curative methods include organ transplantation, surgical resection, percutaneous ethanol injection, and ablation. However, because only a few patients are eligible for surgical resection, radiofrequency ablation (RFA), which is considered safe and has a low mortality rate and mild sequelae, has become a crucial treatment option for primary and secondary liver tumors. In RFA, image-guided techniques such as ultrasound (US) [1,2], computed tomography (CT) [3,4,5], magnetic resonance imaging (MRI) [6,7], and fluoroscopy [8] are frequently used to aid in the localization of treatment sites. US is the most widely used guiding instrument in clinical practice because of its ability to provide real-time images, which accelerates the surgical process without generating any radiation. However, pulmonary obstruction or rib blockage may cause hepatocellular carcinoma (HCC) in the hepatic dome to be partially visible on US images, resulting in incomplete ablation and an increased risk of organ damage. This problem can be resolved through the implementation of CT-guided RFA [9,10,11]. CT guidance (CTG) features precise needle insertion and high resolving power, but it does not provide real-time monitoring, and the insertion procedure requires extensive time, which may lead to pneumothorax or damage to major tissues or organs. In 2019, Kan et al. [12] reported that combined CT/US guidance (CT/US-G) was beneficial for hepatic dome HCC. Prior studies have compared US-guided and CT-guided RFA, but the potential advantage of combining US and CT guidance, especially for tumors in challenging locations (e.g., hepatic dome), remains underexplored. [13,14] This study seeks to address this knowledge gap by systematically comparing the clinical outcomes of US guidance (USG), CTG, and CT/US-G RFA in patients with liver tumor, with a focus on patient survival and procedure-related complications.

## 2. Materials and Methods

### 2.1. Patient Selection

This study was approved by the Institutional Review Board of the Kaohsiung Veterans General Hospital (No. KSVGH20-CT-8-15, approval date: 30 July 2020). We collected data on 1322 RFA treatment sessions conducted between January 2008 and November 2019 from the hospital’s picture archiving and communication system. After excluding 210 sessions not involving liver tumor treatment, 5 sessions related to liver transplantation (as transplantation fundamentally alters long-term survival and thus limits comparability), and 125 sessions with follow-up periods of less than 3 months (to ensure sufficient outcome observation), a total of 982 RFA sessions performed on 553 patients were included in the final analysis. According to the size and location of the liver tumor lesion, patients may undergo either a single RFA session or multiple treatment sessions at the discretion of the treating physician. We then categorized these patients into a multiple-treatment group (*n* = 205) and a single-treatment group (*n* = 348), which comprised 634 and 348 treatment sessions, respectively. We further divided each group into three subgroups depending on the guiding instruments used in their sessions. The total numbers of the CTG, CT/US-G, and USG treatment sessions were 464 (multiple-treatment group = 321, single-treatment group = 143), 246 (multiple-treatment group = 154, single-treatment group = 92), and 272 (multiple-treatment group = 159, single-treatment group = 113), respectively.

### 2.2. Investigations Before RFA

Before RFA, a series of tests, including a complete blood count, hepatic and renal function tests, prothrombin time, and electrocardiogram, were conducted to determine whether the patients met the RFA treatment criteria. Both US and CT were performed 1 day before RFA to determine the size and location of the liver tumor lesions. When preoperative imaging revealed multiple lesions requiring multiple treatment sessions and a tumor size greater than 3 cm, the patients were preferentially assigned to receive RFA under CTG or CT/US-G, depending on tumor visibility and accessibility.

### 2.3. Percutaneous RFA Technique

All procedures were performed by operators with extensive experience in RFA: Dr. Liang (15 years of experience), Dr. Huang (12 years of experience), and Dr. Chiang (9 years of experience). RFA was performed using an RF Generator-M2004 (RF Medical, Geumcheon-gu, Republic of Korea) with an oscillation frequency between 350 and 500 kHz, 17–22-gauge probes, and a 15 cm long single electrode (RF Electrodes Big-Tip; RF Medical). A color Doppler US (GE LOGIQ E9 XDclear 2.0; GE Healthcare, Munich, Germany) was then used for guidance during the US and combined CT/US-G RFA procedure. A 16-row spiral CT (Somatom Sensation; Siemens Medical Solutions, Munich, Germany) was also used for guidance during the CTG and combined US/CT-G RFA procedure.

### 2.4. USG Percutaneous RFA

Depending on individual treatment requirements and sites, each patient was asked to lie on their back or side on an examination table. A US scan was then performed to confirm the location of the tumor and the site, angle, and depth of insertion. Next, the treatment site was disinfected and covered with a sterile surgical towel. A conductive pad was then attached to the patient’s skin, usually on the thigh, to create an electric circuit. In some cases, certain restraints were used depending on the treatment requirements to prevent interference. Local anesthesia was then induced by injecting 10 mL of 2% xylocaine intradermally near the liver. Once the preparation process was complete, a Chiba needle (Cook Medical, Bloomington, IN, USA) was inserted at the treatment site with USG. After the insertion angle was verified, an RFA needle electrode was inserted through the Chiba needle to ensure precise insertion into the targeted lesion site. The needle electrode was then connected to a power supply to generate heat at its uninsulated tip to create a burn, which required 12 ± 6 min and resulted in a necrotic area measuring 3–5 cm. Analgesics were administered during treatment as appropriate to reduce pain. To ensure complete ablation of the liver tumor, the temperature, frequency, and duration of burning were determined by the size of the tumor and the efficacy of treatment.

### 2.5. CTG Percutaneous RFA

Depending on individual treatment sites, each patient was asked to lie on their back or side on an examination table for a CT scan to locate their tumor. CT images were then used to determine the location, angle, and depth of needle insertion. In these images, tumor conditions were used to determine the frequency of burning and the location of needle placement if multiple burns were required. Subsequently, local anesthesia was induced by injecting 10 mL of 2% xylocaine intradermally. A Chiba needle (Cook Medical) was then used to secure the position of electrode insertion. If changes to the insertion angle were required, another CT scan was performed to verify the orientation of the needle. Once the angle was confirmed, an RFA needle electrode was inserted through the Chiba needle until it reached inside the tumor. Multiple CT scans were typically performed to confirm the location of electrode insertion. Once the needle electrode was in the intended position, it was connected to an RF power supply to begin the ablation treatment. If the lesion was large or if multiple tumors were present, the treatment process was repeated, either by pulling the Chiba needle out by several centimeters and adjusting the needle angle and location inside the liver or by reverifying the insertion location and inserting another needle. The electrode was then connected to an RF power supply for a second or third ablation treatment. Once the treatment was complete and the needle electrode had cooled, the needle electrode was pulled back to the Chiba needle to perform tract ablation and prevent hemoperitoneum, and finally the Chiba needle was pulled out of the body.

### 2.6. Combined CT/US-G Percutaneous RFA

To prevent damage to the main arteries and the bile duct, each patient was asked to lie on their back or left side on an examination table to ensure that the surgeon had a clear view of the needle insertion site. US and CT scans were then performed to confirm the location, path, and angle of needle insertion. Pain relief was achieved by injecting 50–100 mg of flurbiprofen axetil intravenously and locally administering 5–10 mL of 2% lidocaine. An incision with a diameter of 0.5–cm was then made in the skin at the site of insertion. However, the locations of the liver tumors were only partially visible on the US and CT images. Therefore, USG was first used to insert a needle electrode near the tumor, and then CTG was used to further direct the electrode to the center of the tumor. Subsequently, a CT scan was performed to ensure the precise location of the electrode. Finally, burning was performed in a similar manner as in the USG- and CTG-based RFA procedures.

### 2.7. Assessment of Clinical Outcomes

RFA treatment of liver tumors requires continuous follow-up, which can be conducted either using contrast agent injections for CT or MRI assessments to determine the survival and recurrence rates of malignant tumors. A common practice is to perform these assessments 1 month after RFA treatment. If recurrence is observed, then another RFA treatment must be scheduled. However, if no sign of recurrence is observed, then the patient must be asked to make regular follow-up visits every 3 months. Follow-up examinations can be performed using US, CT, or MRI.

An alternative monitoring marker in follow-up examinations is α-fetoprotein (AFP), which is a glycoprotein. Because 70–90% of patients with primary HCC present high levels of AFP, AFP is viewed as an early marker for HCC. AFP also increases in patients with liver cancer, ovarian, or testicular germ cell cancer, and certain types of gastric cancer. It may also increase in pregnant women and in patients with certain benign diseases, such as cirrhosis, acute hepatitis, and chronic active hepatitis. However, AFP-based screening for liver cancer has a sensitivity of only 60%. A patient whose post-RFA AFP values exceed 10 ng/mL and who demonstrates an increasing trend in three consecutive follow-up examinations is considered to be at a risk of recurrence.

### 2.8. Statistical Analysis

All statistical analyses were conducted using IBM SPSS Statistics version 20.0 (IBM, Armonk, NY, USA). A life table was used to review the patients’ survival rates. Kaplan–Meier and chi-square tests were conducted to examine differences in the survival curves of the USG, CTG, and CT/US-G groups. Analysis of variance was then used to compare the average treatment time required in the three groups, and a Bonferroni post hoc test was conducted to confirm the differences between these groups. Logistic regression was used to determine the correlation of complication development with guidance method and treatment time, with the significance level set at *p* < 0.05. To account for multiple comparisons, Bonferroni correction was applied to the *p*-values derived from the regression analysis.

## 3. Results

### 3.1. Baseline Characteristics of Patients

The study sample comprised 982 RFA sessions conducted on 533 patients, including 369 men (66.7%) and 184 women (33.3%). The patients had an average age of 65.7 ± 11.3 years (range: 21–92 years). Of all RFA treatment sessions, 737 (75.1%) and 245 (24.9%) were conducted for HCC and metastatic liver tumors, respectively. The origins of the metastatic liver tumors included the colon, breast, stomach, and ovaries, with 157 (16%) RFA sessions conducted for tumors from the colon. Although the three guidance approaches nonsignificantly differed in terms of original cancer site (*p* = 0.635), they significantly differed in terms of tumor size, tumor number, complication development, and average treatment time (*p* < 0.01). CTG RFA sessions were performed on significantly larger tumors (≥3 cm in 30% of CTG vs. 22% of USG), and more frequently on patients with multiple lesions, which coincided with a higher complication rate (14% for CTG vs. 3.7% for USG, *p* < 0.01) and a longer mean treatment time (87.5 min vs. 56.7 min, *p* < 0.001)—see Table 1—whereas CT/US-G RFA sessions were performed more frequently on patients with multiple lesions (39% for CT/US-G vs. 15% for USG vs. 22% for CTG); however, no significant difference was observed between USG and CT/US-G in terms of complication development (*p* = 0.518) or between CTG and CT/US-G in terms of treatment time (*p* = 0.208; Table 1).

### 3.2. Overall Survival Rate

**Figure 1:**
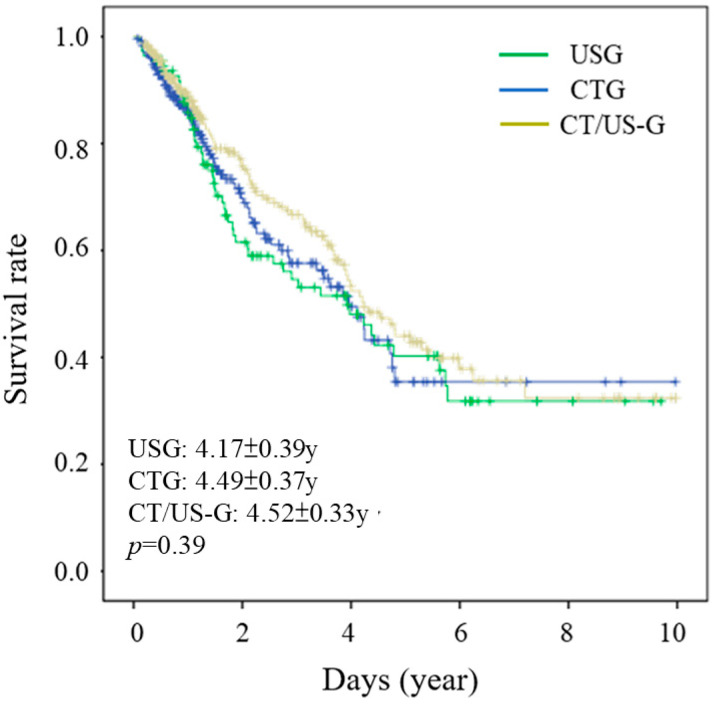
Survival rates of patients who received RFA treatment with USG, CTG, and CT/US-G.

The survival rates of the 533 patients were 84%, 51%, and 35% in the first, third, and fifth year, respectively, after their RFA treatment. The average survival times of patients who received CTG, USG, and CT/US-G were 4.49 ± 0.37, 4.17 ± 0.38, and 4.52 ± 0.33 years, respectively, with the respective median survival times being 3.6 ± 0.51, 2.9 ± 0.75, and 3.8 ± 0.34 years, respectively. However, these biostatistics revealed no significant differences between the three guidance methods in terms of the survival rates of the patients (*p* = 0.39; Figure 1).

### 3.3. Effect of Guidance Method on Complication Development

A comparison of the effect of CTG to USG on complication development revealed an odds ratio (OR) of 3.000 (*p* = 0.03) for single-lesion treatment and 4.941 (*p* = 0.04) for treatment of tumors with a diameter of ≥3 cm in the single-treatment group. In the multiple-treatment group, the OR for CTG vs. USG was 4.232 (*p* = 0.008) for single-lesion treatment and 3.420 (*p* = 0.03) for treatment of tumors with a diameter of <3 cm. A comparison of CT/US-G to USG revealed nonsignificantly different frequencies of complication development regardless of the number or size of tumors in both the single-treatment and the multiple-treatment groups (*p* > 0.05). A comparison of CTG to CT/US-G revealed an OR of 4.333 (*p* = 0.04) for single-lesion treatment and 4.353 (*p* = 0.02) for tumors with a diameter of ≥3 cm in the single-treatment group. In the multiple-treatment group, the OR was 3.853 (*p* = 0.03) for single-lesion treatment and 3.053 (*p* = 0.04) for tumors with a diameter of <3 cm (Table 2).

### 3.4. Effect of Treatment Time on Complication Development

When USG was used to perform a single-lesion treatment, increased treatment time resulted in higher probabilities of complication development (OR = 1.037, *p* = 0.03). When CTG was used to perform a single treatment, the OR of treatment time to complication development was 1.027 (*p* < 0.001) for single-lesion treatment and 1.032 (*p* = 0.008) for treatment of tumors with a diameter of ≥3 cm. When CTG was used to perform multiple treatments, the OR of treatment time to complication development was 1.014 (*p* = 0.01) for single-lesion treatment, 1.022 (*p* = 0.04) for multiple-lesion treatment, and 1.022 (*p* < 0.007) for treatment of tumors with a diameter of <3 cm. However, when CT/US-G was used, no significant difference was observed in the regression model for both the single- and the multiple-treatment groups, suggesting that its treatment time did not affect complication development (*p* > 0.05; Table 3).

## 4. Discussion

Surgical resection and liver transplantation are regarded as an optimal treatment option for liver cancer. However, an increasing number of meta-analytical studies have indicated that, in liver tumors with a diameter of <3 cm, RFA has a similar 5-year survival rate to that of surgical resection, in addition to shorter hospital stays and lower complication rates [15,16,17,18].

El-Serag et al. [19] compared the performance of commonly used liver cancer treatments and discovered that the treatment outcome of RFA was comparable to that of surgical liver resection and liver transplantation. Wu et al. [20] reported that the 1-, 3-, and 5-year survival rates of patients following USG-based RFA were 85%, 70%, and 68%, respectively, and that their respective rates following CTG-based RFA were 84%, 72%, and 58%, respectively, with no significant difference between the two guidance methods (*p* > 0.05). A network meta-analysis by Hu et al. [21] demonstrated that for patients meeting the Milan criteria, RFA yielded 5-year survival rates similar to those of surgical resection. Additionally, studies comparing ultrasound-guided (USG) and CT-guided (CTG) RFA have shown no statistically significant differences in 1-, 3-, and 5-year survival outcomes, suggesting that both modalities are effective and safe for image-guided RFA in clinical practice [22,23]. A comparison of the present study with the extensive review of Wong et al. [24] on RFA revealed that the 1-, 3-, and 5-year survival rates reported in the present study (84%, 51%, and 35%, respectively) were slightly lower than those reported in previous research (Table 4). Further analysis suggested that the patients included in the present study did not conform to the Milan criteria, namely, to have a single tumor with a diameter of ≤5 cm or up to three liver tumors with diameters of ≤3 cm. However, most of the patients included in previous studies met the Milan criteria, meaning that these patients may differ from those included in the present study in terms of the number and size of tumors. This study did not have any exclusion criteria based on tumor number, with the maximum number of tumors in the sample being five and the maximum size being 7.9 cm. In addition, the 5-year survival rate reported in the present study was calculated from the beginning of RFA treatment, which may explain the slightly lower survival rate compared with those reported in previous studies, which included patients who received RFA as their initial cancer treatment. However, the outcomes of the present study are comparable to those of Tateishi et al. [25] in terms of liver cancer recurrence. The present study also remains consistent with those of recent large-scale clinical studies that included real-world populations, such as the study by Kaibori et al. [26], which evaluated RFA outcomes in elderly and advanced HCC cases. Therefore, patients with all tumor sizes, tumor types, and physiological conditions were included in the present study to ensure that the results reflect the actual efficacy of RFA in Taiwan, which was found to be similar to that reported in previous research.

Among the study sample, complications developed in 95 sessions, accounting for 9.67% of all RFA sessions. However, none of these complications were fatal, and most patients recovered within 1–2 weeks. Compared with the findings of previous studies, this study revealed a higher frequency of complication development, which may be due to tumor size, needle insertion path, or physiological differences between the patients. Generally, large tumors prolong treatment time and thus increase the risk of complication development, which is consistent with the present study’s results [12]. Table 5 lists the complications that developed following RFA with different guidance methods, namely, abscess, pneumothorax, biliary injury, right upper quadrant pain, fever, gastric injury, hematoma, hemoperitoneum, and other complications. Pneumothorax was the most frequently observed complication with all three guidance methods, with a frequency of 42 for CTG, 4 for USG, and 12 for CT/US-G. According to previous research, the path of needle insertion may increase the risk of complication development, particularly that of pneumothorax, which may be caused by lowered accuracy in needle insertion due to the inconsistent breathing patterns of patients or by an insertion path that extends through the lung. According to the present study results, pneumothorax accounted for 61% of all instances of complication development, and 51 out of 58 cases of pneumothorax (88%) were due to a needle insertion path that extended through the lung. Maeda et al. [31] reported that the incidence of pneumothorax as a complication in 2011–2015 (0.424%) substantially increased from that in 1999–2010 (0.055%) and that 37 out of 48 pneumothorax cases (77.1%) were observed at the same center. In a more recent observation period, said center performed CTG-based RFA on 106 patients with HCC in the hepatic dome. In these RFA procedures, a needle was inserted at the location of the patient’s lung, which posed a high risk for pneumothorax [10]. Therefore, in the present study, pneumothorax was regarded as a foreseeable complication in patients in whom an RFA needle was inserted through their lungs. Overall, the physiological conditions of patients play a major role in the risk of complication development. Therefore, surgeons, nurses, and radiologists should pay extra attention to the vital signs of their patients and the performance of their guiding techniques during RFA treatment.

Fonseca et al. [32] stated that the likelihood of specific complication development may vary with tumor location. This statement is also applicable to image guidance. Specifically, certain tumor locations may be more visible than others when certain guidance methods are used. This notion is consistent with the present study’s finding that the visibility of tumors located at segment 8 of the liver was higher with CTG than with USG. Segment 8 is close to the diaphragm and ribs, which may cause view obstruction with USG, resulting in low RFA precision with USG. Furthermore, similar to previous research, a relationship between complication development, on the one hand, and treatment method and tumor location, on the other hand, was observed in the present study. Specifically, when RFA was used to treat tumors located in the hepatic dome, the needle was inserted through the lung, which substantially increased the risk of pneumothorax. A tumor located near the veins may also increase the risk of hemorrhage due to a ruptured vein.

Table 6 presents the incidence of complications in each segment of the liver. Complications developed in 95 RFA sessions, with a total of 134 tumors treated in these sessions. When CTG was used, the incidence of complications peaked in treatment cases involving segment 8 of the liver (50.6%). Prior reports have linked pneumothorax to needle paths traversing the lung, especially in patients with inconsistent respiration [10,11]. In the present study, 88% (51/58) of pneumothorax cases were caused by trans-pulmonary needle paths. During the treatment of lesions located in the hepatic dome or close to the diaphragm, complications may occur because the needle may pass through the lung or because the patient may have inconsistent breathing patterns during the treatment process. Maeda et al. [31] also reported a rising pneumothorax rate over time, especially in hepatic dome RFA with CTG.

The outcomes of CT/US-G-based RFA were similar to those of CTG- and USG-based RFA, with CTG-based RFA having the lowest frequency of complication development. Kan et al. [12] reported a nonsignificant difference between the frequencies of complication development following RFA with CT/US-G and CTG (*p* = 0.0224). They discovered that two of the patients who received CTG-based RFA developed pneumothorax, but they observed no complications in patients who received CT/US-G-based RFA. This finding is consistent with that of the present study.

Kan et al. [12] also compared CT/US-G and CTG and discovered that CTG required repeated adjustments for the needle insertion angle and path because of the inconsistent breathing patterns of their patients with each CT scan. Consequently, CTG-based RFA took a longer time compared with CT/US-G-based RFA. In their study, they reported 1-, 3-, and 5-year survival rates of 100%, 97%, and 94%, respectively, following CT/US-G-based RFA. However, their treatment time and survival rates differed from those reported in the present study, which is presumably because their study included 65 patients with a total of 70 tumors. Among these patients, 60 had a single tumor and 5 had two tumors, with each tumor having a diameter of ≤3 cm. In addition, the sample of patients and tumors and the tumor sizes reported by Kan et al. [12] were smaller than those reported in the present study, which may explain why they did not observe any difference between CT/US-G and CTG and why they proposed an increased efficacy of the RFA treatment.

This study has several limitations. First, liver function assessments such as Child–Pugh and MELD scores, as well as standardized performance status data, were not consistently available and thus were not included in the analysis. These clinical parameters are known to influence treatment selection and long-term outcomes, and their absence may limit the interpretability of our findings. Second, the choice of RFA guidance modality (USG, CTG, or CT/US-G) was not randomized but rather determined by tumor characteristics and physician preference, introducing the potential for selection bias—particularly related to tumor size, location, and visibility on ultrasound. Third, the recurrence rates were not analyzed due to the retrospective nature of the dataset and inconsistent follow-up imaging protocols across patients. As a result, we were unable to assess or compare long-term local tumor control among the different guidance groups. These limitations underscore the need for prospective studies with standardized clinical data and follow-up protocols to further clarify the comparative effectiveness of RFA guidance modalities.

## 5. Conclusions

RFA is an effective minimally invasive treatment for liver tumors. However, the accuracy of US-guided RFA may be limited by respiratory motion, rib interference, and poor visualization of tumors near the diaphragm or heart. CT guidance overcomes these limitations by providing consistent anatomical detail, regardless of tumor location. In this study, CTG was associated with the highest complication rate, likely due to larger tumor size and longer procedure time. Interestingly, CT/US-G had a similar treatment duration to CTG but a much lower complication rate. These findings suggest that CT/US-G is a favorable option for treating large, multiple, or difficult-to-access liver tumors, potentially improving safety while reducing radiation exposure.

## Figures and Tables

**Table 1:** Tumor type, tumor size, tumor number, complication development, and average treatment time for USG, CTG, and CT/US-G.

Index	USG	CTG	CT/US-G	*p* Value
Tumor type				0.635
HCC	199	354	184	
Non-HCC	73	110	62	
Tumor diameter				<0.001 *
<3 cm	245	504	317	
≥3 cm	69	218	66	
Number of lesions				<0.001 *
1	231	364	150	
≥2	41	100	96	
Complications				<0.01 ^1,2^
Yes	10	65	20	
No	262	399	226	
Mean treatment time (min)	56.7 ± 21.6	87.5 ± 31.8	90.7 ± 37.8	<0.001 ^3,4^

* Significant difference between USG, CTG, and CT/US-G. ^1^ USG vs. CTG, *p* < 0.01. ^2^ CTG vs. CT/US-G, *p* = 0.007. ^3^ USG vs. CTG, *p* < 0.001. ^4^ USG vs. CT/USG, *p* < 0.001.

**Table 2:** ORs of CTG vs. USG, CT/US-G vs. USG, and CTG vs. CT/US-G for complication development.

Odd Ratio (95% Confident Intervals)	Single Treatment	Multiple Treatments
CTG vs. USG		
Single lesion	3.000 (1.142–7.878) (*p* = 0.03)	4.232 (1.457–12.293) (*p* = 0.008)
Multiple lesions	N/A *	N/A **
Tumor diameter < 3 cm	2.884 (0.883–9.421) (*p* = 0.08)	3.420 (1.159–10.096) (*p* = 0.03)
Tumor diameter ≥ 3 cm	4.941 (1.031–23.689) (*p* = 0.04)	N/A **
CT/US-G vs. USG		
Single lesion	1.719 (0.529–5.583) (*p* = 0.36)	1.098 (0.24–5.034) (*p* = 0.90)
Multiple lesions	N/A *	N/A **
Tumor diameter < 3 cm	2.337 (0.598–9.129) (*p* = 0.22)	1.120 (0.244–5.142) (*p* = 0.88)
Tumor diameter ≥ 3 cm	1.135 (0.191–6.730) (*p* = 0.88)	N/A **
CTG vs. CT/US-G		
Single lesion	1.745 (0.655–4.649) (*p* = 0.26)	3.853 (1.145–12.936) (*p* = 0.03)
Multiple lesions	4.333 (1.029–18.257) (*p* = 0.04)	0.908 (0.32–2.572) (*p* = 0.85)
Tumor diameter < 3 cm	1.234 (0.404–3.771) (*p* = 0.71)	3.053 (1.017–10.424) (*p* = 0.04)
Tumor diameter ≥ 3 cm	4.353 (1.324–14.315) (*p* = 0.02)	1.474 (0.581–3.736) (*p* = 0.41)

N/A: Not applicable. * Sample size too small for analysis. ** Sample size = 0.

**Table 3:** ORs of treatment time to complication development for USG, CTG, and CT/US-G.

Odd Ratio (95% Confident Intervals)	Single Treatment	Multiple Treatments
USG		
Single lesion	1.037 (1.002–1.074) (*p* = 0.03)	1.007 (0.969–1.047) (*p* = 0.71)
Multiple lesions	N/A *	N/A **
Tumor diameter < 3 cm	1.035 (0.981–1.092) (*p* = 0.21)	1.014 (0.966–1.064) (*p* = 0.58)
Tumor diameter ≥ 3 cm	1.033 (0.97–1.099) (*p* = 0.31)	N/A **
CTG		
Single lesion	1.027 (1.012–1.042) (*p* < 0.001)	1.014 (1.003–1.025) (*p* = 0.01)
Multiple lesions	1.038 (0.996–1.083) (*p* = 0.08)	1.022 (1.0–1.044) (*p* = 0.04)
Tumor diameter < 3 cm	1.031 (0.998–1.065) (*p* = 0.06)	1.009 (0.991–1.028) (*p* = 0.33)
Tumor diameter ≥ 3 cm	1.032 (1.008–1.057) (*p* = 0.008)	1.022 (1.006–1.038) (*p* = 0.007)
CT/US-G		
Single lesion	1.011 (0.983–1.040) (*p* = 0.44)	1.009 (0.979–1.040) (*p* = 0.56)
Multiple lesions	1.018 (0.992–1.045) (*p* = 0.16)	1.014 (0.994–1.034) (*p* = 0.17)
Tumor diameter < 3 cm	1.022 (0.996–1.048) (*p* = 0.10)	1.001 (0.953–1.051) (*p* = 0.98)
Tumor diameter ≥ 3 cm	1.007 (0.984–1.030) (*p* = 0.55)	1.014 (0.995–1.033) (*p* = 0.15)

N/A: Not applicable. * Sample size too small for analysis. ** Sample size = 0.

**Table 4:** Comparison of survival rates with previous studies.

Study	Patient No.	Max Size (cm)	Major Complication (%)	Survival Rate (%)			Median Survival (Month)
				1 Year	3 Year	5 Year	
Xu et al. [16]	622	≤3	4.1	93	72	49	54.8
Yue et al. [22]	198	≤5	9.1	82	50	29	32.4
Yu et al. [23] (US-guided)	45	≤5	6.7	89	64	N/A	N/A
Yu et al. [23] (CT-guided)	45	≤5	8.9	86	60	N/A	N/A
Wong et al. [24] (RFA)	136	5	6.1	94	61.1	40.3	N/A
Wong et al. [24] (OP)	97	8	26	89	59.5	47.4	N/A
Tateish et al. [25]	319	5	4	95	78	54	N/A
Kaibori et al. [26]	1500–2000	≤3	4–8	90–95	65–75	45–55	50–60
Lencioni et al. [27]	206	5	2	97	67	41	57
Choi et al. [28]	570	5	1.9	95	70	58	77
N‘Kontchou et al. [29]	235	5	0.9	NA	60	40	48
Han et al. [30]	152	≤5	7.2	89	53	35	39
Maeda et al. [31]	9411	N/A	2.9	95	70	50	N/A
Our study	553	7.9	9.7	84	51	35	47.6

N/A: no data available.

**Table 5:** Frequencies of complications with USG, CTG, and CT/US-G.

Complications	USG	CTG	CT/US-G
Abscess	1 (10%)	2 (3%)	1 (5%)
Pneumothorax	4 (40%)	42 (64%)	12 (60%)
Biliary injury	2 (20%)	1 (2%)	0 (0%)
RUQ pain and fever	0 (0%)	3 (5%)	0 (0%)
Gastric injury	1 (10%)	1 (2%)	0 (0%)
Hematoma	1 (10%)	5 (7%)	4 (20%)
Hemoperitoneum	0 (0%)	1 (2%)	1 (5%)
Hemothorax	1 (10%)	10 (15%)	1 (5%)
Other	0 (0%)	0 (0%)	1 (5%)
Total	10	65	20

**Table 6:** Incidence of complications with USG, CTG, and CT/US-G in each liver segment.

Complication Position	USG	CTG	CT/US-G
S1	0 (0%)	3 (3.4%)	0 (0%)
S2	0 (0%)	5 (5.7%)	2 (5.4%)
S3	3 (30%)	2 (2.3%)	1 (2.7%)
S4	1 (10%)	8 (9.2%)	8 (21.6%)
S5	2 (20%)	2 (2.3%)	5 (13.5%)
S6	1 (10%)	12 (13.8%)	4 (10.8%)
S7	1 (10%)	11 (12.6%)	7 (18.9%)
S8	2 (20%)	44 (50.6%)	10 (27%)
Total number of lesions	10	87	37

## Data Availability

The original contributions presented in this study are included in the article. Further inquiries can be directed to the corresponding author.
